# Four concurrent feedforward and feedback networks with different roles in the visual cortical hierarchy

**DOI:** 10.1371/journal.pbio.3001534

**Published:** 2022-02-10

**Authors:** Elham Barzegaran, Gijs Plomp

**Affiliations:** Perceptual Networks Group, Department of Psychology, University of Fribourg, Fribourg, Switzerland; McGill University, CANADA

## Abstract

Visual stimuli evoke fast-evolving activity patterns that are distributed across multiple cortical areas. These areas are hierarchically structured, as indicated by their anatomical projections, but how large-scale feedforward and feedback streams are functionally organized in this system remains an important missing clue to understanding cortical processing. By analyzing visual evoked responses in laminar recordings from 6 cortical areas in awake mice, we uncovered a dominant feedforward network with scale-free interactions in the time domain. In addition, we established the simultaneous presence of a gamma band feedforward and 2 low frequency feedback networks, each with a distinct laminar functional connectivity profile, frequency spectrum, temporal dynamics, and functional hierarchy. We could identify distinct roles for each of these 4 processing streams, by leveraging stimulus contrast effects, analyzing receptive field (RF) convergency along functional interactions, and determining relationships to spiking activity. Our results support a dynamic dual counterstream view of hierarchical processing and provide new insight into how separate functional streams can simultaneously and dynamically support visual processes.

## Abstract 2

Visual stimuli evoke fast-evolving activity patterns that are distributed across multiple cortical areas, but how large-scale feedforward and feedback streams are functionally organized in this system remains unclear. Visual evoked responses in laminar recordings from six cortical areas in awake mice reveal how layers and rhythms dynamically orchestrate functional streams in vision.

## Introduction

Visual processes exhibit complex patterns of fast-evolving activity that are distributed across cortex. Within 100 ms after stimulus onset, activity spreads throughout visual cortex and beyond, both in primates and rodents [1,2], enabling functional cortico–cortical interactions that are necessary for even the most elementary visual operations [3,4]. Such networked processing occurs over dense anatomical projections that reciprocally connect cortical areas. Across visual cortex, the structure of projections indicates a hierarchical organization, with feedforward projections that can propagate sensory activity from lower to higher level areas, and feedback projections that can exert downstream influences [5–7]. Understanding how visual cortex enables fast, distributed processing over its fixed hierarchical structure may provide important clues to further understanding large-scale cortical function.

Visual processing critically depends on both feedforward and feedback processes [8]. Based on onset latencies after stimulation, it was shown that the feedforward spread of activity from V1 mostly follows the structurally defined hierarchy [1,2] and that a fast feedforward sweep precedes feedback signals from higher-order areas [9]. But due to the fast mixing of activities after onset, it remains unclear how feedforward and feedback interactions dynamically shape visual processing, which mostly occurs after activity onset.

Candidate mechanisms for how visual cortex could support a mixture of feedforward and feedback processes have been proposed based on the laminar structure of hierarchical projections and the rhythmicity of neural activity. Recordings across cortical columns have indicated laminar and frequency differences in feedforward and feedback activities [4,10], where gamma activity in supragranular layers is thought of as feedforward propagation [10–13], and lower frequency activity in infragranular layers is associated with interareal feedback [14,15]. But, although quasiperiodic activity is pervasive in neural systems and much studied, there is no strong consensus on the functional role of different frequency bands [15–17]. In addition, scale-free activity without a dominant rhythm is an equally widespread phenomenon in neural systems [18,19].

To functionally characterize feedforward and feedback streams in the time and frequency domain, we used visually evoked activity recorded in awake mice across the visual hierarchy with laminar resolution. By using Parallel Factor Analysis (PARAFAC) of directed cortico–cortical networks, we uncovered the simultaneous presence of 2 feedforward and 2 feedback networks, each with distinct laminar connectivity profiles, operational frequencies, temporal dynamics, and functional hierarchical organization. The dominant network had scale-free properties in the time domain, and for each of the 4 processing streams, we could determine distinct roles by leveraging well-described effects of stimulus contrast, analyzing receptive field (RF) convergency [20,21], and determining its relation to spiking activity dynamics across the cortical hierarchy.

## Results

### Neural activity and functional interactions show complex dynamics

**Fig 1:**
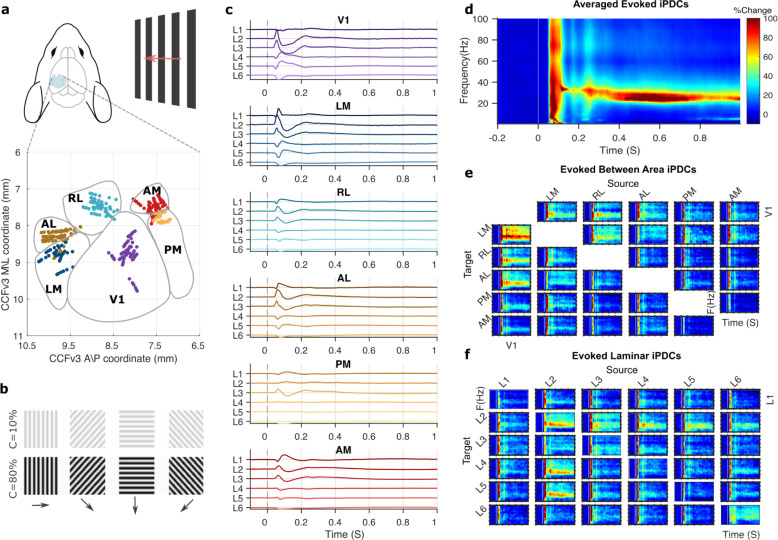
Visual stimuli evoke large-scale activity and functional interactions. **(a)** Laminar recording sites across cortical areas from 11 mice, in the Allen Institute for Brain Science’s standard CCFv3 space. **(b)** Visual stimuli were drifting gratings (2 Hz) of high (80%) or low (10%) contrast, presented for 2 S. **(c)** Grand average bipolar LFPs for high contrast stimuli, for L1 to L6 in 6 visual areas, shown in anatomical hierarchical order [7]. **(d)** Directed functional connectivity (iPDC) in the time and frequency domain, averaged over all areas, layers, and animals. **(e)** Time–frequency connectivity between areas, averaged over layers, and animals. **(f)** Time–frequency connectivity between layers, averaged over areas, and animals. Heatmaps show percentage of poststimulus change following the colormap in **d**, columns indicate sources, and rows indicate targets of directed functional interactions. Underlying data: https://osf.io/pqf7z. iPDC, information partial directed coherence; LFP, local field potential.

We analyzed local field potentials (LFPs) from 11 awake wild-type mice recorded simultaneously from 5 or 6 visual areas with laminar resolution during presentation of high or low contrast drifting gratings (Fig 1A and 1B). These data were made available by the Allen Institute for Brain Science (see Methods). Stimulus-evoked LFPs showed expected fast dynamic responses across layers and areas throughout the cortical hierarchy (Fig 1C) [2], in line with results from nonhuman primates [1].

To derive functional interaction strengths between all areas and layers, we used an optimized Kalman filter to model dependencies between LFPs and calculated the information partial directed coherence (iPDC) [22,23]. iPDC provides a multivariate measure of directed functional connectivity (Granger causality) with high temporal and frequency resolution. It indicates how well activity in each layer predicts activity in all other layers, within and between areas. The resulting directed functional connectivity matrices revealed a detailed time–frequency representation of between-area interaction strengths across visual cortex. After stimulus onset, the overall interaction strengths quickly and transiently increased across the frequency spectrum (Fig 1D). This was followed by sustained interactions in the beta and low gamma band, with a narrowband power reduction around 60 Hz [24,25]. The area-by-area summary showed that fast broadband increases occurred for interaction strengths from all areas, but that outgoing interactions from V1 showed the strongest response, with clearly sustained interactions across time (Fig 1E). The overall laminar profile revealed that the strongest interactions came from L2 (mostly targeting L2, L4, and L5 in other areas), L3 (to L2 and L3), L4 (to L2 and L3), L5 (to L2), and L6 (to L2 and L6), while interactions from L1 were weaker by comparison (Fig 1F).

**Fig 2:**
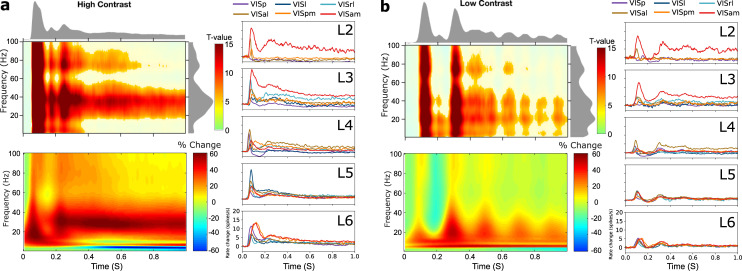
Stimulus-related functional connectivity, PSD, and spiking activity. **(a)** The upper left panel shows time–frequency plots of statistically significant functional connectivity increases with respect to baseline (1-way *t* tests *p* < 0.05, Bonferroni corrected), for high contrast stimuli. Data averaged across layers and areas, the marginal distributions reflect t-values. The lower left panel shows overall changes in PSD (Morlet wavelet) with respect to prestimulus time. The panels on the right show averaged spike rate changes with respect to prestimulus time for L2 to L6 of each area. **(b)** Shows the corresponding results for low contrast stimuli. Underlying data: https://osf.io/pqf7z. PSD, power spectral density.

Averaging across areas and layers, we confirmed that stimulus onset induced statistically robust functional connectivity changes and found that the interactions were contrast sensitive. Induced connectivity onset was around 50 ms for high contrast and 85 ms for low contrast stimuli (Fig 2), following known contrast dependency of LFPs [20]. The initial response was followed by sustained, frequency-specific increases in gamma band interactions that peaked around 35 and 25 Hz for high and low contrast stimuli, respectively. This indicates that reducing stimulus contrast lowers the gamma band interaction frequency, similar to its effect on LFP power spectra [16,26,27].

The overall power spectral density (PSD) in the time and frequency domain showed an important resemblance to the overall iPDC results. For high contrast stimuli, PSD showed a quick broadband increase in power that was followed by sustained activity in the beta and lower gamma band. For low contrast stimuli, a rhythmic modulation of power in these bands was apparent, together with induced power in the theta band. The area- and layer-specific PSD for low and high contrast stimuli are provided in S1 Fig. These supplementary PSD data also further illustrate the previously observed narrowband 60-Hz power decreases and their relation to stimulus contrast [24,25]. Resemblance between iPDC and PSD in the time–frequency domain is expected, because power in an area is necessary (although not sufficient) for it to exert casual influence onto another area (in both the physical and statistical sense of causal), and it confirms that interactions occurred together with induced spectral power in the source regions [28].

Using the corresponding spike-sorted data from the Allen Institute for Brain Science [2], we confirmed that neuronal firing rates reflect key aspects of the functional connectivity and spectral power dynamics. Averaged spiking activity showed robust responses shortly after stimulus onset, in all cortical areas and most layers (Fig 2). Spiking activity showed typical early onsets in L4 of V1 and a quick temporal progression of activity across areas and layers [2]. In addition, rhythmic activity was apparent across layers for low contrast stimuli induced, and it appeared to be more synchronized across areas in the infragranular layers [29]. These results confirm that the iPDC and PSD data are supported by local spiking activity that follows a similar dynamic across layers and areas. We next leveraged the directionality of the iPDC results by investigating the 2-way functional interactions between all layers and areas.

### Four concurrent networks in mouse visual cortex

To further analyze the time- and frequency-resolved functional connectivity across visual cortex, we used PARAFAC, a tensor rank decomposition method that provides robust and interpretable results [30]. PARAFAC has previously been used to identify constituent components of time-varying PSD [31,32] and functional connectivity [33]. PARAFAC guarantees a unique solution when the appropriate number of components is selected. To select this number, we combined multiple criteria, including the core consistency diagnostic, mean square error, model convergence, and visual inspection [30,32,34]. This showed that the functional connectivity matrices were best decomposed into 4 components, independently for low and high contrast conditions, and explaining around 90% of total variance. We confirmed the reliability and validity of the 4-way model across animals using a bootstrap analysis that showed high consistency across bootstraps for each component (average correlation coefficients for all components > 0.81, *p* < 0.05) and low correlations between components (average correlation coefficients = 0.21, not significant).

**Fig 3:**
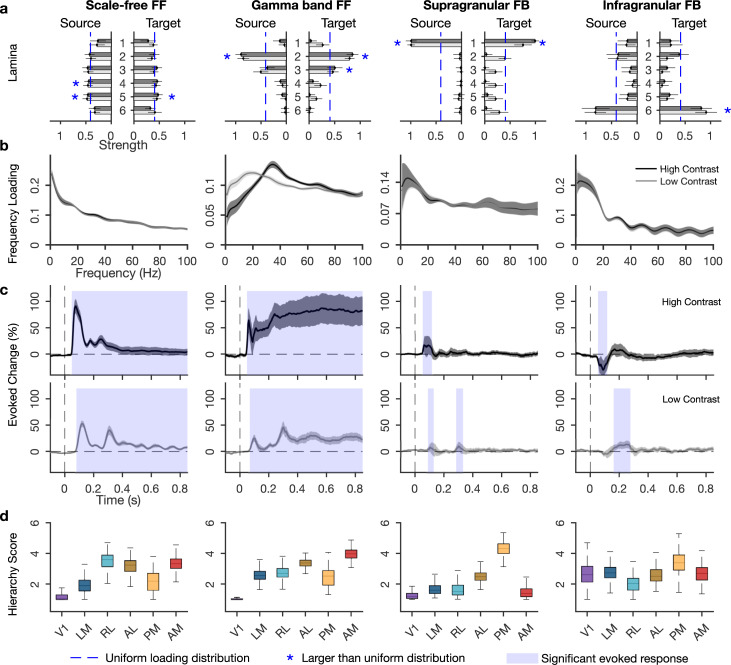
Four concurrent functional networks in visual cortex. **(a)** Laminar input and output strengths (loadings) for the 2 FF and 2 FB networks. Blue stars indicate layers with larger amplitude than the uniform distribution, calculated based on 95% confidence interval. **(b)** Frequency distributions of each network. **(c)** Temporal dynamics per network, as percentage of change compared to prestimulus period, calculated from temporal loadings. Shading indicates statistically significant increases from baseline. **(d)** Functional hierarchy scores calculated from between-area connectivity loadings (see Methods). The higher and lower limits of box plots indicate bootstrapped 25 and 75 quantiles of the hierarchy scores, and whiskers indicate extreme values excluding outliers. For a, b, and c, error bars and gray shading indicate SDs over bootstraps (*n* = 500). Underlying data: https://osf.io/pqf7z. FB, feedback; FF, feedforward.

This way, we established that the dynamic laminar functional connectivity pattern across the visual hierarchy can be decomposed into 4 distinct networks, each of which reflects a distinct processing stream defined by its directed connectivity strengths between areas over source layers, target layers, frequency spectrum, and temporal dynamics (Fig 3).

### A dominant scale-free network

We found that a network with scale-free properties in the time domain was the dominant network, accounting for about 50% ± 5% (mean ± SD) of the PARAFAC model amplitude, in both stimulus contrasts. The connectivity strengths in this network were relatively uniformly distributed over source and target layers, but showed significantly stronger connections from L4 and L5 and toward L5 than expected by chance (Fig 3A). This pattern of laminar connectivity resembles the distribution of feedforward targeting neurons through infragranular layers, particularly L5, as established by retrograde tracing in mouse [7] and monkey [6].

The network’s frequency spectrum followed a power law distribution for both low and high contrast stimuli, revealing a scale-free temporal dynamic where no particular rhythm dominates the functional interactions (Fig 3B). Power law distributions take the form 1/f^β^, where *β* quantifies the self-similarity in the time domain (autocorrelation). Model fitting of the scale-free network showed a *β* exponent of 0.4, lower than typical autocorrelations for electrophysiological data, which fall in the range of 1 to 3 for the frequency range of 1 to 100 Hz [19]. To test whether the low *β* values are characteristic of the network or of the underlying LFPs, we fitted power law distributions to the PSDs of LFPs in all areas. This revealed higher *β* exponents (2 ± 0.5 mean ± SD over bootstraps), demonstrating that low self-similarity rather is a property of the scale-free network than of the underlying LFPs. We note that the iPDC calculation involved a normalization that was done independently for each time and frequency point, which avoids systematic effects of 1/f from the LFP. These results indicate that between-area functional interactions have greater agility in time than the within-area activity.

The scale-free network showed robust amplitude increases quickly after stimulus onset, which decreased after around 150 ms, but exceeded prestimulus values throughout the epoch (*p* < 0.05, Bonferroni corrected, Fig 3C). This fast network activation, together with its laminar profile, supports the idea that sensory activation is quickly relayed across areas over infragranular layers [35]. The scale-free network showed different dynamics depending on stimulus contrast. With low contrast, onset latencies were delayed (from around 50 to 80 ms), and a more pronounced second peak occurred at around 300 ms before amplitudes decreased (cf Fig 2).

To characterize whether the direction of interactions followed a predominant feedforward or feedback pattern, we derived functional hierarchy scores per area, using the asymmetry of between-area functional connectivity strengths [12] (see Methods). Each area was assigned a level in a functional hierarchy, such that the net functional interactions went from lower to higher level areas. By comparing the functional hierarchies to the structural hierarchy established from axonal projections [7], we inferred the predominant flow direction. For example, when the functional hierarchy follows the structural hierarchy, this indicates a feedforward flow since structurally defined low-level areas are driving higher level areas more strongly than the other way around. Vice versa, when structurally defined high-level areas are at the bottom of the functional hierarchy, this suggests a feedback network because high-level areas more strongly drive lower-level areas.

The scale-free network showed a functional hierarchy with V1 and LM at the bottom followed by the other areas (Fig 3D), indicating a mainly feedforward direction of interactions. The functional hierarchy resembled the structural hierarchy established from axonal projections [2,7], but it put PM at a lower ordinal position. Area PM, like V1, preferentially responds to low temporal and high spatial frequencies, while LM, AL, RL, and AM prefer high temporal and low spatial frequencies [36]. The temporal and spatial frequencies of the drifting gratings were closer to the preferred frequencies of PM, allowing it to more strongly drive activity in other areas, thus lowering its position in the functional hierarchy.

In summary, we found a dominant scale-free network whose laminar connectivity profile and functional hierarchy indicate a predominant feedforward direction of interactions. The network responds strongly but transiently to stimulus onsets with some contrast sensitivity.

### Visual stimuli recruit a feedforward gamma band network

The second strongest network contributed 18% ± 2% of total model amplitude, for both stimulus contrast. Its laminar connectivity pattern showed L2 and L3 as both the strongest source and target layers (Fig 3A), a pattern that is typically associated with feedforward processing streams in mouse and primate [6,37–39].

This network showed clear peak amplitudes in the gamma band, broadly defined as 25 to 100 Hz. Visually induced gamma band activity is typically linked to feedforward processes [11,26] and may help synchronize activities between cortical regions [13]. The network’s peak frequency changed with stimulus contrast, centering around 38 Hz for high contrast and around 26 Hz for low contrast (Fig 3B), resembling the frequency shift observed in overall functional connectivity strengths (Fig 2), and the contrast sensitivity of gamma band LFP responses [16,27]. Such strong contrast sensitivity indicates a role in processing stimulus content.

The gamma band network also showed contrast dependency in its temporal dynamics (Fig 3C). For high contrast stimuli, this network showed increased amplitudes after 50 ms that were sustained throughout stimulus presentation. In the low contrast condition, amplitude increase started around 70 ms and were sustained with an apparent rhythmic modulation. These effects resemble the contrast effects in the scale-free network, as well as those observed in overall functional connectivity, spectral power, and spiking activity. The specific rhythmicity with low but not high contrast stimuli makes it unlikely that the induced rhythm simply reflects the grating temporal frequency (2 Hz).

Functional hierarchy analysis confirmed the feedforward character, with V1 located lowest and the ordinal position of the other areas following the structural hierarchy [7,38]. As in the scale-free network, the gamma band network showed a relatively low hierarchical position for area PM, possibly due to the stimulus characteristics and response properties of this area [36].

In sum, the gamma band network shows a laminar interareal connectivity pattern and hierarchical organization that conforms to known feedforward structural connectivity patterns. The network showed a sustained response to visual stimulation that strongly depended on stimulus contrast.

### Two low frequency feedback networks

In addition to the feedforward networks, we uncovered 2 feedback networks that accounted for 17% ± 2% and 15% ± 4% of model amplitude. One was a supragranular network, with putative L1 as the main source and target of interareal connections, and the other was an infragranular network with L6 as the main source and target (Fig 3A). Anatomical studies of primate visual cortex in monkey established that besides the well-known feedback pathway over L6, another feedback pathway exists over supragranular layers L1 and L2 [6,38,39]. Our findings for the first time distinguish these 2 feedback pathways using analysis of functional data and demonstrate their presence in mouse.

In accord with a presumed feedback function, we found that both networks showed a broad low frequency distribution, which peaked at 5 and 6 Hz, respectively, and included the alpha band (full width half maximum of 1 to 14 Hz and 2.5 to 15.5 Hz, respectively). Previous works identified low frequency interactions as signatures of feedback processing in visual cortex of cat and monkey, using functional connectivity analysis and causal interference [10,40], although theta has also been associated with feedforward processes [12,41].

The 2 feedback networks showed strikingly different response dynamics and contrast sensitivities. The supragranular feedback network showed fast and transient amplitude increases with typical contrast sensitivity, i.e., slower onset and reduced amplitudes for low contrast stimuli (50 versus 85 ms). In addition, low contrast stimuli induced a second amplitude increase at around the same latency that the feedforward networks showed a second low contrast peak. The infragranular feedback network, conversely, showed quickly decreased amplitudes for high contrast stimuli, between 50 and 120 ms, but increased amplitudes for low contrast stimuli at longer latencies, between 155 and 280 ms. This indicates enhanced feedback for low contrast stimuli at longer latencies [9,21]. In addition, these findings show how stimulus contrast differentially recruits the 2 feedback pathways, first over infragranular and then over supragranular pathways, suggesting a functional dissociation of feedback streams.

The feedback networks had distinct functional hierarchies. The supragranular network showed AM and V1 at the bottom of a hierarchical organization that otherwise resembled the structural hierarchy [2,7], but with lower hierarchy scores for RL and AL than in the feedforward networks. The low hierarchical positions of high-level visual areas characterize the supragranular network as a feedback network, in line with its laminar connectivity and frequency spectrum. For the infragranular network, area RL showed the lowest hierarchy score, but the other areas appeared not to be hierarchically ordered, with V1 having a higher hierarchy score than in any of the other networks. The low RL position supports a predominant feedback directionality for the infragranular network.

Taken together, these findings distinguish 2 feedback networks, 1 operating over supragranular and 1 over infragranular layers. The networks share a low frequency profile but differ in how they respond to stimulus onset, their contrast sensitivity, and their functional hierarchies. This differentiation is in line with the distinct influences of feedback arriving in the superficial or deep layers and how this affects neural activity in the cortical column [35,42–44].

### Rhythmic modulation of feedforward networks

**Fig 4:**
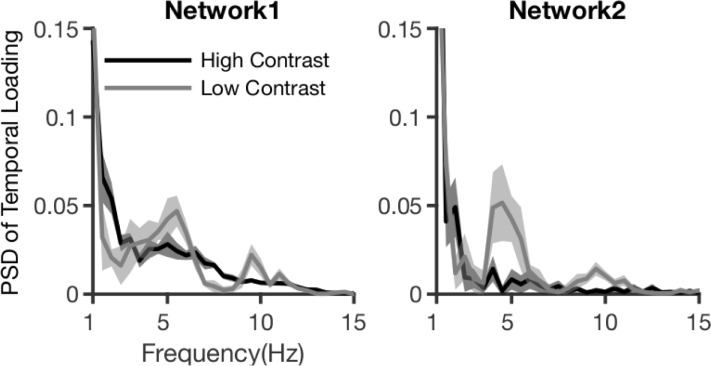
Spectral power of FF network dynamics. Fourier transformation of temporal dynamics for FF networks, for 2 contrast conditions. Shaded area indicates SD over bootstraps. Underlying data: https://osf.io/pqf7z. FF, feedforward.

The feedforward networks appeared to show rhythmic amplitude modulations in time, especially for low contrast stimuli (Fig 3). These modulations were similar to the rhythmicity observed in PSD, spiking activity dynamics (Fig 2, S1 Fig), and bipolar LFPs (S2 Fig). To further investigate this modulation, we applied Fourier transformation on the poststimulus window of temporal loadings, after removing linear trends. This revealed a rhythmic modulation of both feedforward networks that was most prominent for low contrast stimuli and had a peak frequency of around 5 Hz (Fig 4).

Theta band rhythms in mice are common in hippocampus, but also occur in sensory areas [15,45]. In the current data, the theta modulation is likely of cortical origin and unlikely to result from micro-saccades, because of how it varies with stimulus contrast. The possibility that accounts for the theta modulation of feedforward networks was excluded in a control analysis. Theta rhythms in sensory cortex have been shown to organize local activity, in mouse and monkey [41,45,46]. Although theta rhythmic functional interactions were previously reported in monkey [47] and cat [14], our results show a rhythmic amplification of entire feedforward networks, particularly when stimuli are less visible.

### RF distances and functional connectivity

Considerable evidence shows that feedforward projections tend to converge onto matching RFs in upstream areas, whereas feedback projections tend to be more divergent [6,8,48]. Convergent and divergent projections play different roles in spatial processing, and we therefore asked how interareal functional connectivity strengths related to the distance between RF centroids in the source and target location. We hypothesized that functional connectivity strengths would increase with RF overlap for feedforward, but not for feedback networks.

**Fig 5:**
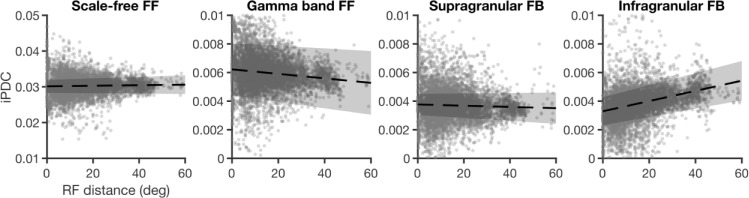
Connectivity as a function of RF distance. Dots represent values across bootstraps, dashed lines represent the regressed line, and the shaded areas indicate 95% confidence intervals (on intercept and slope of the model). Data from high contrast condition. Underlying data: https://osf.io/pqf7z. RF, receptive field.

To test this, we modeled functional connectivity strength as a function of distance between RF centroids for all recording locations (areas and layers), using linear mixed-effect models with bootstrap as a factor (see Methods). This confirmed the convergent nature of interactions for the gamma band feedforward network (${p<0.001,\;R}_{adj}^{2}=0.88$), but not for the scale-free network (${p=0.26,\;R}_{adj}^{2}=0.76$) (Fig 5). In addition, this analysis uncovered a divergent pattern of interactions for the infragranular feedback network, with stronger interactions toward more spatially distinct RFs (${p<0.001,\;R}_{adj}^{2}=0.89$). No RF dependence was observed for the supragranular feedback network (${p=0.16,\;R}_{adj}^{2}=0.7$). These results reveal spatial processing differences between the feedforward and feedback networks by showing how the spatial organization of RFs in the source and target location codetermine functional interaction strengths.

### Spiking activity is tied to feedforward networks

Our network results were based on bipolar LFP signals, which reflect an aggregate of synaptic and postsynaptic currents across the recorded neuronal population (here: 80 μm of cortex) [49,50]. It is well established that LFPs often correlate with spiking activity, particularly in the gamma band [10,49], but how neuronal activity relates to functional networks across visual cortex remains unexplored. Establishing the relationship between spiking activity and directed interactions in the visual hierarchy can potentially shed light on what areas and layers are driving network dynamics and what the effects of network activity on local spiking are. To investigate this, we correlated the amplitude dynamics of each of the 4 networks with time series of laminar firing rates. In this analysis, positive correlations indicate that network amplitudes evolve in synchrony with neuronal activity, suggesting that activity drives the network and/or that the network drives the activity to some extent. Negative correlations indicate that neural firing decreases with increased network presence and rebounds with network absence, in line with a possible inhibitory influence of the network.

**Fig 6:**
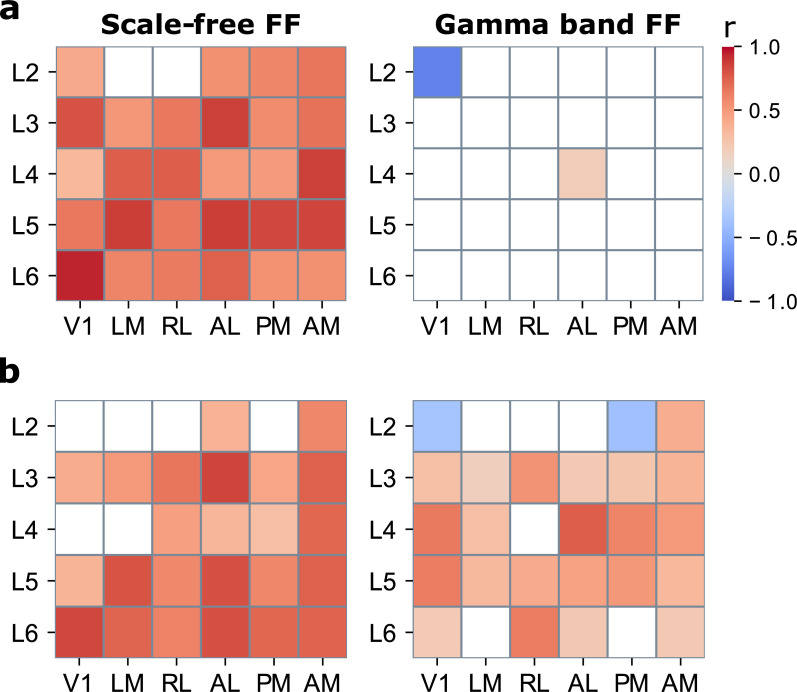
Correlations between network amplitudes and spiking activity. **(a)** Correlation coefficients (*r*) per area and layer for high contrast stimuli and **(b)** for low contrast stimuli. Colored areas indicate statistically significant correlations at *p* < 0.05, 2-tailed bootstrapped distribution. L1 was excluded for lack of spiking activity. Underlying data: https://osf.io/pqf7z. FF, feedforward.

The results showed statistically significant positive correlations between the scale-free network and local spiking activity (*p* < 0.05, 2-tailed bootstrapped distribution), in all areas and almost all layers (28/30 areas and layers; *r* = 0.67 +/− 15) for high contrast stimuli (Fig 6). In addition, the gamma band network correlated positively with activity at L4 of area AL (*r* = 0.19) and negatively with L2 of V1 (*r* = −0.75). No significant correlations with the putative feedback networks were observed. For low contrast stimuli, spiking activity showed statistically significant positive correlations between with the scale-free network, at 24/30 sites (*r* = 0.61 +/− 17). In addition, widespread positive correlations were found for the gamma band network, at 22/30 sites (*r* = 0.41 +/− 17). This suggests that low contrast stimulation enhances the link between neuronal activity and feedforward gamma band interactions across the cortical hierarchy. Negative correlations appeared with L2 of V1 (*r* = −0.37), as observed for high contrast stimuli, and L2 of area PM (*r* = 0.36). The supragranular network, furthermore, positively correlated with activity in L4 of V1 (*r* = −0.38), in line with the idea that feedback enhances weak inputs [9,21]. The infragranular network showed negative correlations with activity in L5 of V1 and PM (*r* = −0.42 and −0.49, respectively), possibly through inhibition of activity by way of feedback interactions over infragranular layers [51].

These results reveal a close link between spiking activity and the presence of the scale-free network. They extend the previously established influence of spiking activity on scale-free LFP signals [52] by showing that the dynamics of neuronal activity synchronize with scale-free feedforward interactions across visual cortex. By contrast, the feedback networks appeared not to be closely linked to neuronal firing. This suggests that feedback network dynamics are not synchronized with activity dynamics, which may have been expected given the relatively weak presence of feedback in these data.

## Discussion

We found that functional interactions across visual cortex can be reliably decomposed into 4 constituent directed networks, based on their distinct laminar connectivity profiles, operational frequencies, temporal dynamics, and hierarchical organizations. The laminar connectivity pathways match anatomical knowledge and provide strong functional evidence for the simultaneous presence of multiple feedforward and feedback streams [6,37] and extend the notion of functional hierarchies in cortex [2]. We furthermore show distinct functional roles for each of these streams in response to stimuli, reconciling the hierarchical structure of visual cortex with its fast distributed processing.

Our analyses demonstrate the existence of 2 distinct feedforward processing streams. Previous studies have associated feedforward sensory processing with gamma band activity in supragranular layers [10,12], in line with anatomical connectivity patterns of these layers [6,38]. Our gamma band network provides strong functional support for a feedforward role for layers 2/3 pathways mediated by gamma band interactions. But in addition, our results indicate a second feedforward stream over infragranular layers that has a scale-free frequency distribution. Evidence of an infragranular feedforward pathway has been previously reported based on anatomical data [6], but, to our best knowledge, it has not been reported in functional studies.

Our results identify distinct roles in visual processing for the scale-free and the gamma band network. An important clue to the possible function of the scale-free network lies in its power law frequency distribution. Previous works have related scale-free LFP spectra to overall neuronal population activities [52], but at a larger scale, power law distributions can indicate that a complex system is working near criticality, i.e., at an equilibrium point between 2 states [18]. Dynamics near a critical point can allow neural activity states to rapidly adjust to changes in the external environment, by providing a flexible midlevel synchronization between neuronal rhythms and fast transition between states via phase resetting [53]. In our data, the scale-free network showed the strongest amplitudes and widespread correlations with spiking activity, suggesting its relative importance and a possible role in facilitating the state and activity changes of other frequency-specific networks in response to visual stimulation. This interpretation is supported by its strong but transient response to visual stimuli and its lack of RF convergence. In line with such a dynamic resetting role, the scale-free network also showed low autocorrelation in time and nonconvergent RF projections of its upstream interactions, indicating nonspatial processing.

The gamma band network, by contrast, responded to visual stimuli with a sustained amplitude increase throughout stimulus presentation. This suggests a role in processing stimulus content that is supported by its sensitivity to stimulus contrast, both in the time and frequency domain. In addition, the RF convergence of its interactions make it suited to process spatial stimulus aspects, because convergent interactions preserve spatial position while propagating induced activity up the hierarchy. The peak frequency and amplitude of the induced gamma oscillations are previously reported to be modulated by stimulus properties such as contrast, size, and location [11,16], as well as by selective attention [54,55]. Although gamma band interactions occur in feedback too [55], it has been shown that salient or attended stimuli induce faster and stronger gamma oscillations in early visual areas, and when competing gamma oscillations exist within low-level areas, the faster oscillations have a higher chance to induce activity in higher level areas through phase coding [13,27]. Therefore, the stimulus sensitivity of gamma oscillations has been described as an implementation of bottom-up attentional selection [13]. In our results, the contrast sensitivity of gamma peak and amplitude indicate that elementary forms of such an effect might exist in mice.

We found strong evidence for a theta band modulation of the feedforward networks. This theta modulation seems unlikely to be caused by the feedback networks, because they did not show a sustained response, whereas the theta modulation persisted over the whole period of stimulus presentation (Fig 4). Given its contrast dependency, a cortical origin seems the most likely. Cortical theta activity in sensory areas has been linked to feedforward interactions [12,17] and plays a role in rhythmic perceptual sampling in monkey and human [46,56]. A proposed mechanism of perceptual sampling is based on theta–gamma cross-frequency coupling, where theta modulates feedforward activity through resynchronization of gamma oscillations between lower and higher visual areas by resetting of the gamma phase [13,41]. In line with this, when the gamma band feedforward network showed a theta band modulation in response to low contrast stimuli, its correlation with spiking activity was also increased. In future work, investigating spike–LFP coherence could test whether local spike synchronization plays a role in this process [50]. Our results show that entire feedforward networks can be modulated by a theta rhythm and provide first evidence for a rhythmic visual sampling in mouse. If behaviorally verified, this means that rhythmic visual sampling is an evolutionary well-preserved feature and that the causal investigations possible in mice can be employed to help further understand this phenomenon.

To best estimate directed functional interaction strengths from the nonstationary LFP dynamics, we derived multivariate Granger causality indices (iPDC) using time-varying MVAR models based on an optimized Kalman filter [23]. While we took utmost care in the modeling, the limits of functional connectivity analysis apply. Granger causality is a statistical measure that identifies systematic relations between recorded signals, which inform hypotheses about neural function [57,58]. While LFP signals reflect a mixing of outgoing and incoming synaptic activity, both of within- and between-area projections, using full multivariate modeling with a high number of recording sites brings functional connectivity estimates closer to physiological reality [23,50,59]. In addition, and in contrast to other coherence measures, iPDC is insensitive to power imbalances between signals [22]; therefore, our finding of gamma band feedforward and low frequency feedback is unlikely to result from power gradients across the cortical hierarchy [50].

We uncovered 2 feedback networks that operate at alpha and theta frequencies [10,12]. While the theta band is not exclusively tied to feedback functions [17,41,60], the laminar and hierarchy results indicate that these 2 networks are more in line with a feedback than a feedforward role. Moreover, each feedback network showed distinct laminar connectivity, functional hierarchy, contrast sensitivity, and relationship with RF distances (Fig 3), suggesting a functional dissociation between feedback streams for stimulus-dependent spatially organized feedback and global modulatory feedback [8]. The infragranular feedback stream showed a particular contrast sensitivity, with fast amplitude decreases for high contrast, and slower but amplitude increases for low contrast stimuli. In addition, its connectivity pattern was spatially organized, with stronger connections to locations with nonoverlapping RFs. This organization is compatible with a role in contextual spatial processing, such as surround suppression and figure–ground segmentation [4,10,48], operating over infragranular layers in stimulus-specific ways [61]. It has been shown that feedback over infragranular layers can affect local processes in the cortical column through upward propagation [35,42]. Likewise, feedback at supragranular layers can modulate activity throughout the column [42]. In our data, the supragranular feedback stream showed no spatial organization of connections, and less sensitivity to contrast, making it suitable for global or nonretinotopic feedback modulations [62]. In line with this characterization, the lowest areas in its hierarchy were motion-sensitive areas AM, LM, and RL [36].

The supragranular network showed L1 as a feedback source. In functional connectivity terms, this means that L1 LFPs predict activity in lower-level areas. In mouse and primate, L1 is an important target of feedback projections; it contains few, mostly inhibitory, neurons and sends few extrinsic projections [38,63]. LFPs at L1 thus mainly reflect input from other areas, which can modulate activity throughout the cortical column and the column’s output to other areas [38,42–44]. Since the data used here did not include cortical areas beyond AM, we cannot exclude the possibility that another area accounts for the observed functional feedback connection from L1, e.g., by projecting to AM but also to lower-level areas with some delay. Another possibility is that L1 signals partly reflect activity from L2, a known feedback source, due to tissue compression from electrode insertion. With the current data, we can neither exclude these possibilities nor conclusively link the putative L1 feedback role to physiology.

Both feedback networks showed a transient low frequency response that peaked in the theta band (Figs 2C and 4), and while theta is typically observed in feedback [10,40], it has also been associated with feedforward processes [46,60]. Likewise, gamma band activity and connectivity changes are taken to reflect feedforward processes [11,26], but have been observed in feedback as well [55]. This means that theta band spectra in themselves are not fundamental to feedback, just as gamma is not fundamental to feedforward communication. Therefore, the labeling of a network as feedforward or feedback cannot be based on frequency spectra alone. We therefore used additional criteria based on directed network analysis, including laminar interaction pathways, hierarchy analysis, and sensitivity to stimulus properties. Even then, we do not conceive of processing streams as either strictly feedforward or feedback, but rather as situated on a continuum between 2 theoretical extremes. For the infragranular network, for example, the laminar connection pattern resembles anatomical feedback projections that were previously established, and, although the functional hierarchy is not an inverted feedforward one, the fact that area RL is at the bottom of the hierarchy speaks in favor of a feedback interpretation, as does the network’s enhanced presence with low contrast and the absence of RF convergence. Taken together, this characterizes the network as a predominantly feedback stream, and this characterization helps us better understand what the transient low frequency response constitutes and what its possible role in vision is.

Feedback processing is generally thought to arise after a first feedforward sweep, in a 2-stage process [9]. Our results show partial support for such a sequential view, in that infragranular feedback increased at longer latencies (150 ms), and selectively for low contrast stimuli where feedback is expected to play a greater role [21]. In addition, however, our results show that stimuli can evoke quick feedback processing over the supragranular stream and that they can also quickly suppress ongoing feedback processes. Suppression of theta and alpha feedback due to attention has been previously reported [41]. Taken together, our results characterize visual responses as a dynamic reweighting of feedforward and feedback processes, which are better separable in terms of their functional hierarchies, laminar connectivity, and operational frequencies, than they are in time.

Overall, feedback amplitudes were small compared to the feedforward amplitudes [39], and while feedback was transient, feedforward networks showed more robust responses, for longer time, and with widespread correlations to spiking activity. Anatomical studies in monkey show that feedback projections are more numerous than feedforward projections, but that feedforward projections are stronger, especially at shorter range [6]. Our results confirm this with functional evidence and characterize the induced response as a predominantly feedforward process. But even though the stimuli and task were not optimized for investigating typical roles attributed to feedback connections, like figure–ground segregation [4,21], contour integration [3] or selective attention [55], the demonstrated presence of 2 feedback streams in awake but passive mice points to a fundamental importance for feedback in vision.

Our results provide support for the counterstream model of hierarchical processing, as derived from primate anatomy [37,39]. Hierarchical projections follow a similar architecture in rodents and primates, but there are important differences. One difference is that laminar segregation of feedforward and feedback pathways appears less stringent in mice than in primates [63,64]. In line with this, we found that the scale-free network has a relatively homogeneous laminar target and source pattern (Fig 2). We can hypothesize the laminar differentiation of scale-free feedforward connectivity to be more pronounced in primates, should similar functional analyses become possible there. An additional difference is that L6 is known to project to L1 in primates, but this projection seems weak or absent in mice [7]. The infragranular network showed sources and targets in L6, without targeting L1, and therefore resembles mouse anatomy more than primate anatomy. Since the presence of a structural connection is necessary but not sufficient for a functional interaction, it may depend on the task whether a L6–L1 functional interactions can be demonstrated. Overall, however, our results corroborate the similarities between the large-scale functional organization of processing streams in mouse and primates, which opens up new avenues for investigating large-scale cortical function using causal manipulations.

## Methods

### Dataset and visual stimuli

We used publicly available recordings from the Visual Coding—Neuropixels dataset, provided by the Allen Institute for Brain Science [2]. This dataset contains LFPs recorded simultaneously in 4 to 6 visual areas of awake mice, using Neuropixel probes [65] (40 μm distance between recording channels), with 2.5-KHz sampling rate. Full details on surgery, stimulation protocols, and recording techniques are available in the technical white paper “Allen Brain Observatory–Neuropixels Visual Coding,” 2019 (portal.brain-map.org/explore/circuits/visual-coding-neuropixels). The data were downloaded in the format of raw Neurodata Without Borders (NWB), using Allen SDK 1.2.0 (2019). The LFPs, spike-sorted data, and metadata can be accessed via the AllenSDK, by following the instructions on this page: https://allensdk.readthedocs.io/en/latest/visual_coding_neuropixels.html.

We used a subset of the Brain Observatory stimulus set with a large number of trials, the Functional Connectivity stimulus set. From 14 available LFP recordings in wild-type mice, we selected 11 for analysis (average age = 126 ± 10 days, 10 males) that showed clear source and sink patterns of current source density (CSD) and had simultaneous recordings from at least 4 separate areas (Fig 1A). These areas were V1 (recordings from 11 animals), AL [10], RL [9], PM [7], LM [6], and AM [11] of the left hemisphere; 2 animals had simultaneous recordings in 6 areas, 6 in 5, and 3 in 4.

From the standardized battery of visual stimuli [66], we selected drifting gratings for their reliably evoked LFP signals and high number of trials (75 trials for each grating orientation and contrast). Gratings were presented in random order, with a high (80%) or low (10%) contrast at detectable levels [67], 4 different orientations (0, 45, 90, or 135 degrees), and with a spatial frequency of 0.04 cycles/deg and temporal frequency of 2 Hz (drifting left to right). Stimuli were presented monocularly to the right eye on a monitor that covered 120 × 95 degrees of visual angle. The paradigm consisted of 2 seconds of stimulus presentation and 1 second of interstimulus interval during which a uniform mean luminance gray was presented.

### Preprocessing and layer assignment

For each trial, we considered the epoch from 300 ms before to 1,000 ms after stimulus onset. We pooled the epochs from 4 orientations together, which resulted in 300 epochs per animal, per contrast.

To identify representative electrodes for each cortical layer (L1 to L6) we derived CSD patterns from averaged LFP data [68]. We assigned layers based on sources and sinks across cortical depth, following [45], taking into account depth estimates provided with the dataset. The average cortical depths of selected electrodes for each layer are listed in Table 1.

**Table 1:** Average cortical depth of selected channels for each layer and area (mean ± SD).

	V1	LM	RL	AL	PM	AM
L1	80 ± 25	87 ± 39	76 ± 13	73 ± 24	80 ± 0	80 ± 25
L2	160 ± 25	167 ± 39	156 ± 13	149 ± 31	183 ± 31	164 ± 28
L3	255 ± 32	253 ± 41	244 ± 13	233 ± 35	297 ± 31	269 ± 40
L4	407 ± 39	400 ± 51	360 ± 35	356 ± 52	451 ± 30	407 ± 50
L5	542 ± 55	560 ± 51	511 ± 52	495 ± 60	583 ± 31	545 ± 48
L6	785 ± 70	767 ± 39	733 ± 45	720 ± 82	777 ± 39	764 ± 55

The Allen Institute for Brain Science provides spatially down sampled LFP data, preserving every fourth contact point of the Neuropixel probe, resulting in 80 to 100 units per probe, with 40-μm distance between the available LFP signals. In order to remove the common reference, which could cause spurious estimation of functional connectivity, we applied bipolar rereferencing by computing the difference of the LFPs from 2 neighboring channels [69,70], subtracting activity from electrodes immediately below and above the representative LFP signal for each layer, i.e., over a 80-μm distance. LFP data were downsampled to 250 Hz after anti-aliasing filtering.

### Functional connectivity

To estimate time- and frequency-resolved directed functional connectivity, we used a multivariate parametric approach, the self-tuning optimized Kalman filter (STOK) [23]. STOK uses a Kalman filter formulation that is optimized to model rapidly fluctuating between-signal dependencies under unknown noise conditions, resulting in a time-varying multivariate autoregressive (tvMVAR) model. While nonparametric methods can be used to accurately estimate directed connectivity strengths in the time and frequency domain, when appropriately conditionalized, the optimized Kalman filter is computationally lighter and suffers less from time–frequency trade-offs [23,59,71,72]. To estimate the tvMVAR model in STOK, a model order parameter *p* should be estimated prior to analysis. This parameter indicates how much of the past information should be included in estimation of current state. Here, we selected the optimal model order by minimizing the difference between the tvMVAR model power spectra and the spectra calculated by a nonparametric method based on complex Morlet wavelet [59], for each animal separately. We then selected the maximum optimal order observed over animals (*p* = 15 samples or 60 ms) as the model order and calculated tvMVAR models per animal and condition (low, high contrast) using all single trial epochs and a filter factor of 0.98. STOK code is freely available for MATLAB (https://github.com/PscDavid/dynet_toolbox) and Python (https://github.com/joanrue/pydynet).

After Fourier transformation, tvMVAR coefficients were normalized to express a directed measure of functional connectivity in line with Granger causality, the information partial directed coherence (iPDC) [22,73,74]. Resulting iPDC matrices contained the layer-specific directed functional connectivity between all recorded areas, for every frequency between 1 and 100 Hz (1 Hz resolution) and time point between −300 to 1,000 ms around stimulus onset (4-ms resolution). We preserved the between-area connections for further analysis, averaged across animals, and unfolded the resulting connectivity matrix into 5 dimensions as follows: source layers, target layers, time, frequency, and between-area connections (30 directed connections between the 6 areas).

### PARAFAC

PARAFAC decomposes a multiway matrix into a fixed number of components, with each component represented by a set of loading vectors that correspond to the original data dimensions [30,75]. We applied PARAFAC to the averaged five-dimensional connectivity matrices of absolute iPDC values. The resulting PARAFAC decomposition can be expressed as follows:

$${iPDC}_{sl,tl,t,f,c}=\sum_{k=1}^{K}a_{sl,k}.b_{tl,k}.c_{t,k}.d_{f,k}.e_{c,k},$$

where K is the number of components, and ***a***_***sl*,*k***_, ***b***_***tl*,*k***_, ***c***_***t*,*k***_, ***d***_***f*,*k***_ and ***e***_***c*,*k***_ correspond to the loading vectors for component *k*, for each source layer, target layer, time point, frequency point, and between-area connection. Loading vectors were estimated using alternating least square with random initialization and a nonnegativity constraint. The variance of the data is usually kept in one of the loading vectors of PARFAC model, and the other loading vectors are normalized to have variance of 1. In our case, the variance was kept in the temporal loadings. We used the N-way MATLAB toolbox for PARAFAC decomposition [76].

To derive the most informative and valid PARAFAC model, an appropriate number of components needs to be selected. With too few components, the true underlying components of the data cannot be extracted, while with too many, the results will contain correlated components that do not represent the underlying variables. To identify the appropriate number of components, we combined multiple indicators of model quality and visual inspection of the resulting components, following previous works [30–34]. To exclude the possibility that component selection was driven by data from a subset of animals, we first created bootstrapped averages by randomly selecting 8 (out of 11) animals. For each bootstrapped average (*n* = 10), we calculated Corcondia scores, variance explained, mean squared error, and model convergence. Using these scores across bootstraps, we found that models with 4 components were the best choice for our data: They substantially increased model fit as compared to sparser models, while including more components resulted in high correlations between some of them.

Finally, to verify the reliability of the PARAFAC model across animals, we bootstrapped iPDC averages (*n* = 500, random selection as above) and extracted the 4 components for each bootstrap. We checked component consistency across bootstraps by calculating the pairwise correlations between the loading vectors of PARAFAC components, resulting in intra- and intercomponent consistency values.

### Statistical analysis on PARAFAC loadings

To specify the characteristic of each network, we investigated the loading vectors of the PARAFAC models using statistical analysis. To indicate the source and target layers that contribute most to each network, we compared the loading values of each source/target layer over bootstraps to uniform distribution of weights over layers using *t* test. Uniform distribution indicates no laminar specificity for the network, while layers with loading values larger than the uniform distribution have significant contribution to that network.

For temporal loadings, we determined significant response amplitudes from baseline by comparing the temporal loading values of each poststimulus time point with averaged prestimulus temporal loading values, using *t* test and Bonferroni correction. For visualization purpose, we converted the temporal loadings to percentage of change by subtracting and then dividing each temporal loading by its averaged prestimulus loading values.

Since the variance of the data is preserved in the temporal loadings, we indicated the relative overall amplitude of each PARAFAC component by dividing its averaged temporal loading by the sum of averaged temporal loadings of all components.

### Model comparison of frequency distributions

To characterize frequency distribution of the PARAFAC components, we fitted 3 distributions to the frequency loadings in each bootstrap separately. Power law (in the form of *c*. *f*^−*β*^), lognormal (log *N*(*μ*,*σ*)), and exponential (*c*. *e*^*β*.*f*^) distributions were fitted using *nlinfit* function in MATLAB, and the mean squared error for each fitting was calculated. The model with the lowest average mean squared error over the bootstraps was considered as the best model fit.

We also fitted the same models to the power spectrum density of LFPs, calculated from the tvMVAR coefficients, for each bootstrap and each region of interest (ROI) separately and used mean squared error to indicate the best model fit.

### Functional hierarchy analysis

We used loading vectors of between-area connectivity strengths to calculate hierarchy scores, employing a method specifically proposed for directed functional connectivity strengths [12]. First, the directed influence asymmetry index (DAI_ij_) was estimated based on between-area loading vector with element ***e***_***ij***_ indicating the connection from area *j* to *i*.


$${DAI}_{ij}=\frac{e_{ij}-e_{ji}}{e_{ij}+e_{ji}}$$

We scaled DAIs to the range −2.5 to 2.5 in order to allow 6 levels of hierarchy (considering 6 areas). Then, for each target area, we shifted the DAIs from all source areas so that the smallest value was 1. Then we averaged the rescaled and shifted DAIs ($\hat{DAI}$) for each source to estimate the hierarchy score of the source area as $H_{i}=\sum_{j}{\hat{DAI}}_{ij}$.

### RF analysis

The significant RFs were calculated based on a permutation-based method used in [2] and using the strength of stimulus-related LFPs in a Gabor stimuli condition in the dataset. RF distances were calculated as the Euclidean distance between the centroids of the significant RFs. We only considered the between-area connections that had source and target areas with significant RFs (80%).

To examine the relationship between RF distance and functional connectivity strengths, we reconstructed between-area connectivity strengths from the PARAFAC results using Kronecker products of the loading vectors, for each component and bootstrap separately. We then averaged the reconstructed matrix over time, frequency, and layers to obtain a single connectivity value for each between-area connection.

Using linear mixed-effect regression, we modeled connectivity strengths as a function of the continuous predictor RF distance, including bootstrap and between-area connection as random effects over the intercept, to account for between-area and animal variability, respectively. This model was preferred over a simpler fixed-effects model without random effects, as shown by a likelihood ratio test. Modeling was done using the *fitlme* function with maximum likelihood estimation in MATLAB.

### Spiking activity

We used spike-sorted data as provided by the Allen Institute for Brain Science, from the same recording sessions as the LFPs used for network analysis. We collected spikes for each layer and area from units identified on 4 Neuropixel channels above and below the LFP channel used, corresponding to the same 80-μm distance used for bipolar LFP referencing. Only units with signal-to-noise ratio (SNR) >3 were included for analysis. Average spike counts were calculated from stimulus onset until 1 seconds after in bins of 4 ms for each animal, region, layer, and stimulus contrast. This way, firing rates could be reliably extracted in all areas for L3, L4, L5, and L6. Firing rates could not be well determined for L2 of areas LM and RL (both contrasts). L1 showed little spiking activity overall and was excluded from further analysis.

For each network, we correlated the time series of bootstrapped PARAFAC loading (*n* = 500) with the raw values of average spiking activity from the corresponding subsets of animals. This resulted in a distribution of correlation values across bootstraps, for each layer, area, and contrast. To identify statistically significant values, we thresholded correlations smaller and larger than the 2.5% extremes of the bootstrapped distribution, i.e., *p* < 0.05 for a 2-tailed distribution.

### PSD

PSDs of bipolar-referenced LFP signals were calculated using a Morlet wavelet [59], with a padding of 1 and a central frequency of 6 Hz [59], over 2-second epochs centered on stimulus onset. Results were expressed as change to prestimulus power in the −300 to −50 ms window, avoiding contamination by the smoothed out fast induced response.

## Supporting information

S1 Fig Laminar PSDs per area. **(a)** Shows visually induced PSD (Morlet wavelet) with respect to prestimulus time, for high contrast stimuli. **(b)** Shows corresponding data for low contrast stimuli. Underlying data: https://osf.io/pqf7z. PSD, power spectral density.
(PDF)

S2 Fig Grand average bipolar LFPs for low contrast stimuli. Data for L1 to L6 in 6 visual areas. Underlying data: https://osf.io/pqf7z. LFP, local field potential.
(PDF)

## Abbreviations

CSD: current source density
DAI: directed influence asymmetry index
iPDC: information partial directed coherence
LFP: local field potential
NWB: Neurodata Without Borders
PARAFAC: Parallel Factor Analysis
PSD: power spectral density
RF: receptive field
ROI: region of interest
SNR: signal-to-noise ratio
STOK: self-tuning optimized Kalman filter
tvMVAR: time-varying multivariate autoregressive
