# Supplementary material for: Four concurrent feedforward and feedback networks with different roles in the visual cortical hierarchy
Source: PLoS Biol. 2022 Feb 10;20(2):e3001534. doi: 10.1371/journal.pbio.3001534 (PMC8865670; doi:10.1371/journal.pbio.3001534)
Supplement: S1 Fig — (a) Shows visually induced PSD (Morlet wavelet) with respect to prestimulus time, for high contrast stimuli. (b) Shows corresponding data for low contrast stimuli. Underlying data: https://osf.io/pqf7z. PSD, power spectral density. (PDF) [file pbio.3001534.s001.pdf]

Supplementary Information

Four concurrent feedforward and feedback networks with different roles in the visual cortical hierarchy

Elham Barzegaran, Gijs Plomp

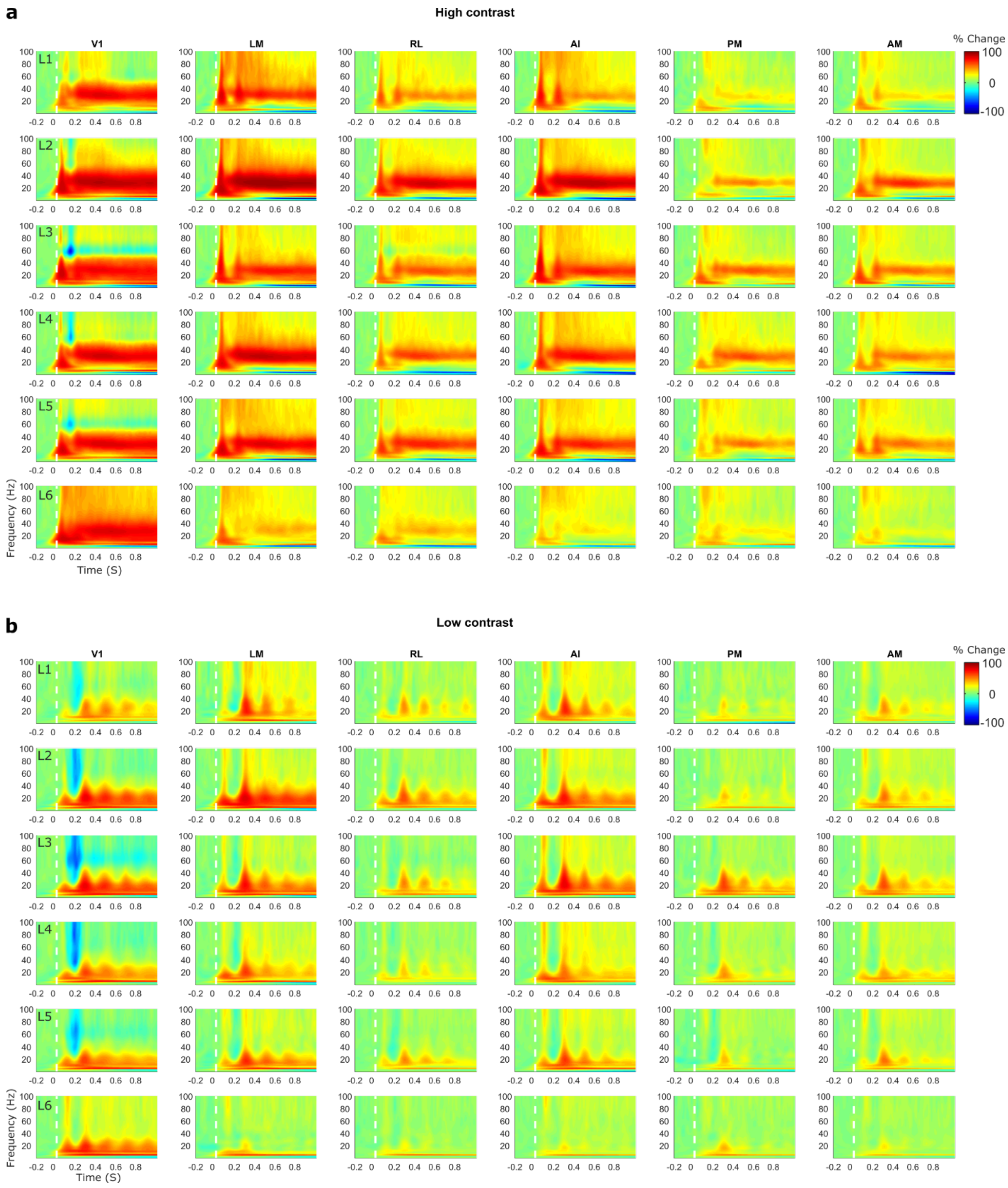

S1 Fig. Laminar power spectral densities per area. a, shows visually induced power spectral density (PSD; Morlet wavelet) with respect to pre-stimulus time, for high-contrast stimuli. **b,** shows corresponding data for low-contrast stimuli. Underlying data: https://osf.io/pqf7z/
