# Supplementary material for: Four concurrent feedforward and feedback networks with different roles in the visual cortical hierarchy
Source: PLoS Biol. 2022 Feb 10;20(2):e3001534. doi: 10.1371/journal.pbio.3001534 (PMC8865670; doi:10.1371/journal.pbio.3001534)
Supplement: S2 Fig — Data for L1 to L6 in 6 visual areas. Underlying data: https://osf.io/pqf7z. LFP, local field potential. (PDF) [file pbio.3001534.s002.pdf]

Supplementary Information

Four concurrent feedforward and feedback networks with different roles in the visual cortical hierarchy

Elham Barzegaran, Gijs Plomp

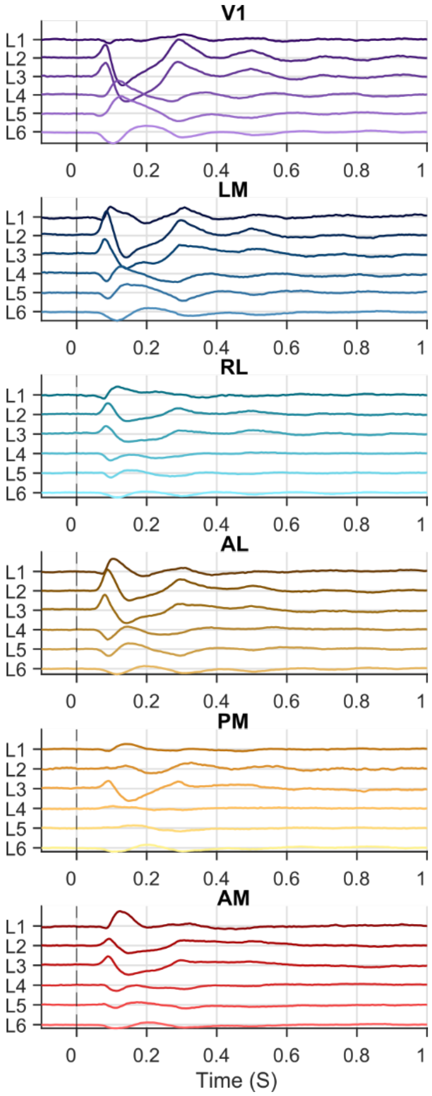

S2 Fig. Grand-average bipolar LFPs for low contrast stimuli. Data for L1 to L6 in six visual areas. Underlying data: https://osf.io/pqf7z/
